# Enitociclib (VIP152), venetoclax and prednisone in relapsed or refractory aggressive non‐Hodgkin lymphoma

**DOI:** 10.1111/bjh.70234

**Published:** 2025-10-30

**Authors:** Max J. Gordon, Rahul Lakhotia, Stefania Pittaluga, Svetlana D. Pack, Anna Marie Juanitez, Ahmed Hamdy, Amy J. Johnson, Melanie M. Frigault, Raquel Izumi, Mark Raffeld, Mark Roschewski, Wyndham H. Wilson, Christopher Melani

**Affiliations:** ^1^ Lymphoid Malignancies Branch, Center for Cancer Research, National Cancer Institute National Institutes of Health Bethesda Maryland USA; ^2^ Laboratory of Pathology, Center for Cancer Research, National Cancer Institute National Institutes of Health Bethesda Maryland USA; ^3^ Vincerx Pharma Inc. San Mateo California USA

**Keywords:** BCL2 inhibitor, CDK9 inhibitor, diffuse large B‐cell lymphoma, high‐grade B‐cell lymphoma double‐hit with *MYC* and *BCL2* rearrangements, peripheral T‐cell lymphoma, targeted therapy

To the Editor,

Dysregulated proliferation and survival are fundamental hallmarks of cancer. In aggressive lymphomas, alterations in the *MYC* oncogene and anti‐apoptotic regulators such as *MCL1* and *BCL2* are frequent promoters of this phenotype.^1^, ^2^, ^3^ MYC is essential for cell growth and proliferation whereas BCL2 and MCL1 regulate cell death and are potentially important therapeutic targets.^4^ Unfortunately, directly targeting MYC or MCL1 has been challenging due to poor clinical activity and excess toxicity.^5^, ^6^ While the BCL2 inhibitor venetoclax has shown modest single‐agent activity in aggressive lymphomas, including diffuse large B‐cell lymphoma (DLBCL) and peripheral T‐cell lymphoma (PTCL), durable remissions are rarely observed.^7^, ^8^

Cyclin‐dependent kinase 9 (CDK9) regulates the activity of RNA polymerase II (RNAPII) and CDK9 inhibition has been identified as a mechanism to indirectly target MYC and MCL1, among other short‐lived proteins, through inhibition of RNAPII transcription.^9^, ^10^ CDK9 inhibitors have shown activity in relapsed or refractory (R/R) lymphomas; however, durable remissions are observed in a minority of patients. In a study of single‐agent enitociclib (VIP152), a CDK9 inhibitor, two of seven patients with high‐grade B‐cell lymphoma double‐hit with *MYC* and *BCL2* rearrangements (HGBCL‐DH‐*BCL2*) achieved complete response (CR), both ongoing after 2.3 and 3.7 years of follow‐up.^11^, ^12^ Increased BCL2 and MCL1 have been identified as mechanisms of resistance to CDK9 and apoptosis, respectively, suggesting a therapeutic benefit to combining venetoclax and enitociclib.^1^, ^13^ Additionally, we have shown that prednisone inhibits B‐cell receptor signalling and is synergistic with venetoclax in aggressive B‐cell lymphoma cell lines.^14^, ^15^ ^,16^ We hypothesized that the combination of enitociclib, venetoclax and prednisone (VVIP) would be active in R/R aggressive lymphomas, many of which express increased *MYC*, *BCL2* and *MCL1*.

We conducted a single‐centre phase 1/2 trial of VVIP in patients with R/R PTCL, *MYC*‐rearranged DLBCL/HGBCL and non‐germinal centre B‐cell (GCB) DLBCL. Patients ≥18 years with adequate organ function were eligible. Two or more prior lines of therapy were required, including prior anthracycline in all patients, brentuximab vedotin in anaplastic large cell lymphoma patients and anti‐CD20 therapy in aggressive B‐cell lymphoma patients. Exclusions included prior allogeneic stem cell transplantation (allo‐SCT) within 6 months of study enrolment, human immunodeficiency virus (HIV) infection, malabsorptive syndrome or significant cardiovascular disease (Supporting Information S1). A ‘3 + 3’ design was used to determine the recommended phase 2 dose (RP2D) of dose‐escalated enitociclib and venetoclax with fixed‐dose prednisone. Primary objectives included determination of the RP2D in the phase 1 cohort and CR rate in the phase 2 cohort with secondary objectives including response rate, response durability, event‐free survival, progression‐free survival (PFS) and overall survival (OS). Phase 2 expansion cohorts of up to 29 patients each were planned for each eligible subtype with a total accrual ceiling of 130 patients.

Four dose levels (DLs) of enitociclib administered intravenously on days 2 and 9 (DL1 = 15 mg, DL2 = 22.5 mg and DLs 3–4 = 30 mg) and venetoclax orally on days 1–10 (DLs 1–3 = 600 mg and DL4 = 800 mg) were given along with fixed‐dose prednisone 100 mg orally on days 1–10 to maximize drug synergy with combination therapy.^15^ ^,16^ Patients received treatment in 21‐day cycles for up to 24 total cycles of therapy. Patients who achieved CR could discontinue treatment after 12 cycles. Pegfilgrastim 6 mg was administered subcutaneously on day 11 (Figure S1). All patients received prophylaxis against *Pneumocystis jiroveccii* throughout treatment in addition to tumour lysis syndrome (TLS) prophylaxis during the first cycle of therapy.

Adverse events (AEs) were assessed using Common Terminology Criteria for Adverse Events version 5.0 and response was determined according to the Lugano Classification Response Criteria.^17^ Computed tomography scans were performed at baseline and following cycle 1 then every other cycle with positron emission tomography at baseline and after cycles 6, 12 and 24. The trial was approved by the Institutional Review Board of the National Cancer Institute and was conducted in accordance with the principles of the Declaration of Helsinki. All patients provided written informed consent prior to study enrolment.

Eight patients enrolled between April 2023 and July 2024 (Table 1; Figure S2). The median age was 56 years (range, 39–79). Five patients had PTCL, two had HGBCL‐DH‐*BCL2* and one had non‐GCB DLBCL. Both HGBCL‐DH‐*BCL2* patients had received prior anti‐CD19 chimeric antigen receptor (CAR) T‐cell therapy. International Prognostic Index score ≥3 was observed in 88% (7/8) and 38% (3/8) had bulky disease ≥10 cm. Median prior lines of therapy were 3 (range, 3–6), including two patients with prior autologous stem cell transplantation, two with prior anti‐CD19 CAR T‐cell therapy and one patient with two prior allo‐SCTs.

**TABLE 1:** Patient characteristics.

Age, gender	Diagnosis	Molecular	IPI	Prior lines	Cellular therapy	Refractory disease
55, M	PTCL, NOS	*BCOR, MAX* ^a^	3	6	Allo‐SCT x2	Y
71, M	PTCL, NOS	*TET2* x2, *SOCS1*	3	3	ASCT	Y
39, M	PTCL, NOS	*FYN:TRAF3IP2* fusion	3	3	ASCT	N
79, M	ALCL, ALK‐	*PIK3R1, ANKRD11, MSC*	4	3	—	Y
77, F	AITL	*RHOA, TET2, DNMT3A* x2, *CTNNB1* ^a^	3	3	—	Y
55, M	HGBCL‐DH‐*BCL2*	*PHF6*	3	5	Anti‐CD19 CAR‐T	Y
48, M	HGBCL‐DH‐*BCL2*	*CREBBP, TP53, SRSF2, ARID1A, CCND3, KMT2D, STAT6, TBL1XR1*	3	4	Anti‐CD19 CAR‐T	Y
56, M	Non‐GCB DLBCL	EZB, MYC‐^b^	2	3	—	N

*Note*: Molecular alterations were identified by whole exome sequencing unless noted otherwise below. Age in years.

Abbreviations: AITL, angioimmunoblastic T‐cell lymphoma; ALCL, anaplastic large cell lymphoma; Allo‐SCT, allogeneic stem cell transplant; ASCT, autologous stem cell transplant; CAR‐T, chimeric antigen receptor T‐cell therapy; DLBCL, diffuse large B‐cell lymphoma; F, female; GCB, germinal centre B‐cell; HGBCL‐DH‐*BCL2*, high‐grade B‐cell lymphoma double‐hit with *MYC* and *BCL2* rearrangements; IPI, International Prognostic Index; M, male; PTCL, NOS, peripheral T‐cell lymphoma not otherwise specified.

^a^ TruSight Oncology 500 (TSO500) targeted sequencing panel.

^b^ LymphGen classification.^2^

Three patients each were treated at DL1 and DL2 and two patients at DL3 for a total of 30 cycles of therapy (median 4 cycles [range, 1–6]). AEs (% patients) observed in ≥2 patients included anaemia (88%), thrombocytopenia (88%), hypokalaemia (88%), neutropenia (75%), hypomagnesaemia (50%), cytomegalovirus (CMV) reactivation (38%; all asymptomatic), diarrhoea (38%), alkaline phosphatase elevation (25%), fatigue (25%) and rash (25%). Three patients had grade (G) 3 AEs (hypokalaemia, thrombocytopenia and neutropenia) and three patients had G4 AEs (all neutropenia). G3–4 neutropenia and thrombocytopenia occurred in 23% and 3% of cycles respectively (Figure S3). No TLS was observed. One dose‐limiting toxicity (DLT) occurred at DL3 (G4 neutropenia ≥7 days) with no other DLTs observed. Two patients had dose delays across three cycles (G3 thrombocytopenia, G2 lung infection and G2 skin infection), and no dose reductions were required. There were no G3–4 infections or treatment‐related deaths. Five patients discontinued treatment due to progression, two at investigator discretion and one due to the requirement of a prohibited concomitant medication (isavuconazole). The RP2D was not determined due to premature study closure triggered by the financial considerations of the drug manufacturer.

Partial response (PR) was observed in 50% (4/8) of patients, including 60% (3/5) with PTCL and 50% (1/2) with HGBCL‐DH‐*BCL2* (Figure 1A). Median time to response was 3 weeks and median duration of response was 3.5 months. With a median follow‐up of 15.5 months, median PFS was 3.6 months overall and 4.1 months in PTCL patients. Median OS was 13.4 months, and three patients died, all due to disease progression (Figure 1B; Figure S4). Two patients with PTCL, not otherwise specified (NOS) received allo‐SCT after stable disease following VVIP and one non‐responding patient with non‐GCB DLBCL received anti‐CD19 CAR T‐cell therapy. All three patients remain alive and progression free.

**FIGURE 1:**
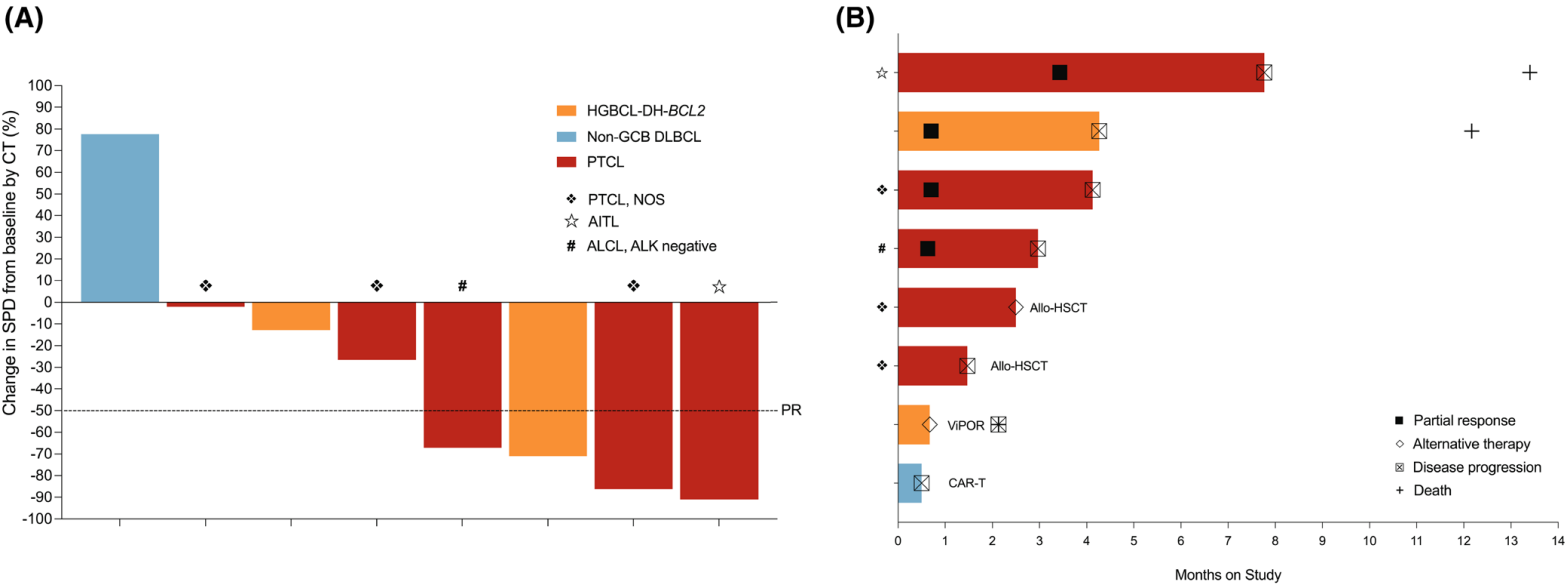
Response and durability to VVIP. (A) Change in tumour burden from baseline, and (B) durability of response and outcome.

Although limited by small numbers and premature study closure, our study demonstrates that VVIP is feasible and active in heavily pretreated, refractory patients with aggressive non‐Hodgkin lymphoma (NHL), particularly PTCL. Overall, the VVIP regimen was associated with expected toxicities, predominantly neutropenia, and the RP2D could not be determined due to premature study closure. Future studies of CDK9 inhibitors with BCL2 inhibition could consider exploring higher doses given the overall favourable safety profile of the doses explored in this study. Despite PRs observed in PTCL and HGBCL‐DH‐*BCL2*, the median duration of response was only 3.5 months, which is consistent with other studies of CDK9 inhibition in R/R lymphomas.^10^, ^11^, ^12^ Importantly, two patients with PTCL, NOS were successfully bridged to allo‐SCT.

Despite the limitations above, the favourable tolerability of the VVIP regimen in addition to its clinical activity suggests that further development of CDK9 inhibitors in combination with other novel agents is warranted. Preclinical studies have demonstrated synergy with CDK9 inhibition in combination with inhibitors of PIM, phosphoinositide 3‐kinase (PI3K) and enhancer of zeste homolog 2 (EZH2).^9^ ^,18^ Given the poor outcome of R/R aggressive NHL, especially PTCL, with standard therapy in addition to the modest single‐agent efficacy of these targeted agents, novel targeted therapy combinations are likely needed to improve overall outcomes in these patients. Better understanding of mechanisms of resistance as well as identifying rational combinations based on preclinical synergy will likely be essential for the future clinical development of CDK9 inhibitors in R/R aggressive NHL.

## AUTHOR CONTRIBUTIONS

M.J.G. treated the patients, analysed the data and wrote the manuscript; R.L., A.M.J. and M.R. treated the patients, analysed the data and reviewed the manuscript; S.P., S.D.P. and M.R. performed the pathology, analysed the data and reviewed the manuscript; A.H., A.J.J. M.M.F. and R.I. conceptualized the study and reviewed the manuscript; W.H.W. and C.M. conceived and conducted the study, analysed the data and helped write the manuscript.

## CONFLICT OF INTEREST STATEMENT

A.H., A.J.J., M.M.F. and R.I. are employees of Vincerx Pharma.

## Supporting information


Data S1.

## Data Availability

The data that support the findings of this study are available on request from the corresponding author. The data are not publicly available due to privacy or ethical restrictions.
