# Supplementary material for: Enitociclib (VIP152), venetoclax and prednisone in relapsed or refractory aggressive non‐Hodgkin lymphoma
Source: Br J Haematol. 2025 Oct 30;208(1):308–11. doi: 10.1111/bjh.70234 (PMC12819095; doi:10.1111/bjh.70234)
Supplement: Supplementary file 1 — Data S1. [file BJH-208-308-s001.docx]

**Supplemental Methods**

## Eligibility Criteria

### Inclusion Criteria:

#### Participants must have a histologically or cytologically confirmed lymphoid malignancy as listed below, confirmed by the Laboratory of Pathology, NCI, as follows:

- - R/R *MYC*-rearranged DLBCL/HGBCL (*MYC* aberration must be confirmed by NCI Laboratory of Pathology to enroll)
  - R/R non-GCB DLBCL without *MYC*-rearrangement (COO and non-*MYC* aberration must be confirmed by NCI Laboratory of Pathology to enroll. COO determination at enrollment will utilize immunohistochemistry and Han’s algorithm)
  - R/R PTCL (PTCL-NOS, PTCL-TFH, follicular TCL, AITL, ATLL, ALK+ ALCL and ALK- ALCL per 2016 WHO classification)

#### Relapsed and/or refractory disease, as defined below:

- - Aggressive B-cell lymphoma: relapsed after and/or refractory to at least 2 prior systemic therapies, 1 or more which includes an anthracycline and anti-CD20 targeting agent
  - PTCL: relapsed after and/or refractory to at least 2 prior systemic therapies, 1 or more which includes an anthracycline (and a brentuximab vedotin-containing regimen for participants with ALK+ or ALK- ALCL)

#### Must have evaluable disease by clinical exam (i.e., palpable lymphadenopathy, measurable skin lesions, etc.), laboratory assessment (i.e., disease involvement of bone marrow or peripheral blood by morphology, cytology or flow cytometry), and/or imaging (measurable lymph nodes, masses, or bony lesions on CT or MRI and/or evaluable FDG- avid lesions on PET).

**NOTE:** Lesions that have been irradiated cannot be included in the tumor assessment unless unequivocal tumor progression has been documented in these lesions after radiation therapy.

#### Age >18 years

#### ECOG performance status <2

#### Adequate organ and marrow function as defined below unless dysfunction is secondary to disease:

| Absolute neutrophil count* | >1,000/mcL |
| --- | --- |
| Hemoglobin* | >8 g/dL |
| Platelets | >75,000/mcL |
| INR | <1.5 X institutional upper limit of normal (ULN) for participants not receiving therapeutic anticoagulation |
| PTT/aPTT | <1.5 X institutional ULN normal except if the aPTT is elevated because of a positive Lupus Anticoagulant |
| Total bilirubin** | <1.5 X institutional ULN (or <3 X institutional ULN for participants with documented Gilberts syndrome) |
| AST(SGOT)/ALT(SGPT)*** | <2.5 X institutional ULN |
| Serum creatinine | < 2.0 mg/dL |
| OR | |
| Creatinine clearance**** | >40 mL/min/1.73 m2 for participants with creatinine levels above 2 mg/dL |
| Cr Cl will be calculated with the use of the 24-hour creatinine clearance or modified Cockcroft-Gault equation (eCCR; with the use of ideal body mass [IBM] instead of mass):  (140 − Age) x IBM (kg) × [0.85 if female] 72 x serum creatinine (mg/dL) | |
| *RBC transfusions and use of G-CSF will be allowed in order to meet eligibility parameters.  **Total bilirubin must be <3 X institutional ULN for eligibility even if secondary to disease.  ***AST(SGOT)/ALT(SGPT) must be <5 X institutional ULN for eligibility even if secondary to disease.  ****Creatinine clearance must be >30 mL/min for eligibility even if secondary to disease. | |

#### Negative serum or urine pregnancy test must be obtained within 7 days before the first dose of study drug in women of childbearing potential. Postmenopausal women, as defined below, are allowed to enroll without a pregnancy test:

- Age >50 years with amenorrhea for at least 12 months or
- Age ≤50 years with 6 months of spontaneous amenorrhea and follicle stimulating hormone (FSH) level within postmenopausal range (>40 mIU/mL) OR
- Permanently sterilized women (e.g., tubal occlusion, hysterectomy, bilateral salpingectomy, uterine ablation)

#### Women and men of reproductive potential must agree to use highly effective contraception when sexually active. This applies for the period between signing of the informed consent and 90 days after the last administration of study drug.

Highly effective contraception includes:

- Established use of oral, injected or implanted hormonal methods of contraception
- Placement of certain intrauterine devices (IUD) or intrauterine systems (IUS)
- Hysterectomy, oophorectomy, salpingectomy or vasectomy of the partner (provided that partner is the sole sexual partner of the woman of childbearing potential trial participant and that the vasectomized partner has received medical assessment of the surgical success)

In addition, participants must agree to use condoms.

#### Participants that are positive for hepatitis B core antibody, hepatitis B surface antigen (HBsAg), or hepatitis C antibody must have a negative hepatitis B and/or C viral load by polymerase chain reaction (PCR), and agree to additional monitoring

#### Ability of participant to understand and the willingness to sign a written informed consent document.

#### Breastfeeding participants must be willing to discontinue breastfeeding from study treatment initiation through 90 days after the last administration of study drug.

### Exclusion Criteria:

#### The following restrictions apply to current or prior anti-cancer treatment, prior to the first dose of study drug:

- - - - - Participants who are actively receiving any other anti-cancer investigational agents.
        - Any chemotherapy, targeted therapy, or anti-cancer antibodies within 2 weeks prior to the first dose of study drug
        - Radio- or toxin-immunoconjugates within 10 weeks prior to the first dose of study drug
        - Prior allogeneic stem cell (or other organ) transplant within 6 months or any evidence of active graft-versus-host disease or requirement for immunosuppressants within 28 days prior to first dose of study drug
        - Not recovered (i.e., ≤ Grade 1 or baseline) from adverse events due to previously administered anti-cancer treatment, surgery, or procedure. **NOTE:** Exceptions to this include events not considered to place the participant at unacceptable risk of participation in the opinion of the PI (e.g., alopecia).

#### Participants requiring the following agents within 14 days or 5 half-lives of the drug (whichever is shorter) prior to the first dose of venetoclax and VIP152 are excluded:

- - - - - Strong CYP3A inhibitors
        - Strong CYP3A inducers
        - Moderate CYP3A inhibitors (dose-escalation cohort only)
        - Moderate CYP3A inducers (dose-escalation cohort only)

**NOTE:** Moderate CYP3A inhibitors and inducers should be used with caution for participants in the dose-expansion cohorts and an alternative medication used, whenever possible.

#### Known allergy to both xanthine oxidase inhibitors and rasburicase; or, known hypersensitivity to any of the study drugs

#### Known active bacterial, viral, fungal, mycobacterial, parasitic, or other infection (excluding fungal infections of nail beds) at study enrollment, or any major episode of infection requiring treatment with IV antibiotics or hospitalization (relating to the completion of the course of antibiotics) within 2 weeks prior to first dose of study drug

#### HIV-positive participants

#### Active CMV infection as determined by a positive CMV PCR

#### Active SARs-CoV-2 infection based on PCR assay; prior SARs-CoV-2 infection allowed if completely recovered from infection and negative PCR testing

#### Clinically significant history of liver disease, including viral or other hepatitis, current alcohol abuse, or cirrhosis; as well as active infection with HBV or HCV

#### Participants with occult (defined as positive total hepatitis B core antibody [HBcAb] and positive HBsAg) or prior HBV infection (defined as positive total hepatitis B core antibody [HBcAb] and negative HBsAg) may be included if HBV DNA is undetectable.

- Participants who are positive for HCV antibody must be negative for HCV by polymerase chain reaction (PCR) to be eligible for study participation

#### Malabsorption syndrome or other condition that precludes enteral route of administration

#### History of other active malignancy requiring therapy that could affect compliance with the protocol or interpretation of results

#### Symptomatic congestive heart failure, unstable angina pectoris, or cardiac arrhythmia

#### Left ventricular ejection fraction (LVEF) < 45%

#### Clinically relevant findings on electrocardiogram (ECG) such as a second- or third-degree AV block or prolongation of the QTc interval (Fridericia) over 470 msec (participants with AV block and pacemaker in place for >1 year and checked by a cardiologist within <6 months before the first dose of study drug, will not be excluded).

#### Uncontrolled intercurrent illness (including psychiatric) or social situations that may limit interpretation of results or that could increase risk to the participant

Dose Limiting Toxicity (DLT) Criteria

A DLT (dose-limiting toxicity) is defined as a grade 3 or higher adverse event (AE) that occurs in the dose-escalation cohort within the first 22 days after initiation of VVIP (i.e., Cycle 1, Day 1 to Cycle 2, Day 1 pre-dose) and is considered related to study drug (i.e., VIP152, venetoclax, and/or prednisone) or a treatment delay of cycle 2 of > 7 days for hematologic or nonhematologic toxicities with the following exceptions:

Non-Hematologic DLT exceptions to above definition

- Grade 3 nausea with maximum medical supportive care and persisting ≤ 7 days
- Grade 3 vomiting or diarrhea if the participant does not require total parenteral nutrition (TPN) or tube feeding, and the toxicity improves to < grade 3 within 72 hours
- Grade 3 fatigue persisting ≤ 7 days
- Grade 3 fever or infection
- Grade 3 rash or dry skin with maximum medical supportive care and persisting ≤ 7 days
- Grade 3 hypo- or hypernatremia, hypo- or hyperkalemia, hypo- or hypercalcemia, hypo- or hypermagnesemia, and/or hypo- or hyperphosphatemia that improves to < grade 3 within ≤ 7 days
- Grade 3 atrial fibrillation that is adequately controlled with medical management

Hematologic DLT exceptions to above definition:

- Grade 3 febrile neutropenia (ANC <1000/mm^3^ with a temperature of >38.3°C [single] or ≥38°C [sustained ≥1 hour]) that is uncomplicated and not associated with infection
- Grade 3 neutropenia (ANC <1000/mm^3^) with maximal G-CSF use persisting for < 7 days
- Grade 4 neutropenia (ANC <500/mm^3^) with maximal G-CSF use persisting for < 7 days
- Grade 4 thrombocytopenia (<25,000/mm^3^) persisting for < 7 days and not associated with grade 2 or greater bleeding
- Grade 3 thrombocytopenia not associated with grade 2 or greater bleeding
- Grade 3 anemia persisting for < 7 days

**Figure S1: VVIP Treatment and Dosing Schema.**

**
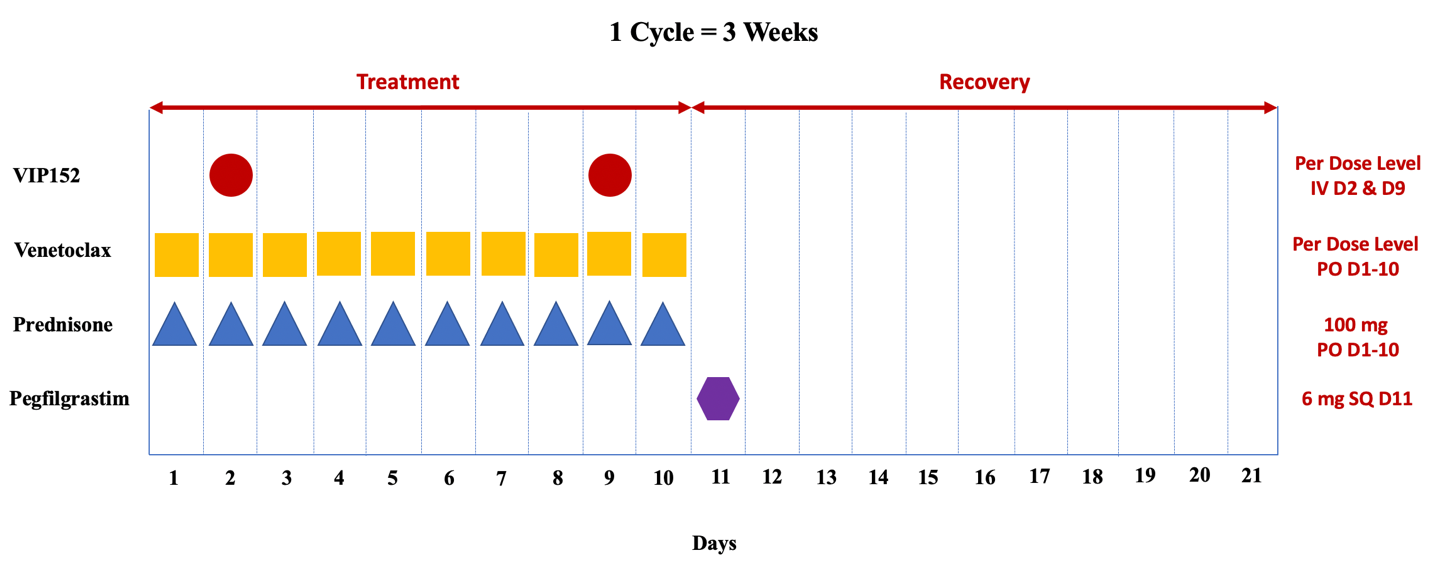
**

| **Dose Level** | **Dose of VIP152** | **Dose of Venetoclax** |
| --- | --- | --- |
| 1 | 15 mg IV on day 2 and day 9 | 600 mg PO daily days 1-10 |
| 2 | 22.5 mg IV on day 2 and day 9 | 600 mg PO daily days 1-10 |
| 3 | 30 mg IV on day 2 and day 9 | 600 mg PO daily days 1-10 |
| 4 | 30 mg IV on day 2 and day 9 | 800 mg PO daily days 1-10 |

**Figure S2: Molecular characterization of tumors.**

| **Pt ID: 55 year old male with PTCL, NOS** | | | | | | | |
| --- | --- | --- | --- | --- | --- | --- | --- |
| **Gene** | **Gene location** | **Transcript** | **Nucleotide change** | **Amino acid change** | **VAF** | **Assessment** | **TIER** |
| BCOR | chrX:39921444 | NM_001123385.2 | c.4376A>G | p.Asn1459Ser | 67% | Pathogenic | Tier 3 |
| MAX | chr14:65543324 | NM_002382.5 | c.353C>A | p.Ser118* | 35% | Likely Pathogenic | Tier 3 |
| **Pt ID: 71 year old male with PTCL, NOS** | | | | | | | |
| TET2 | chr4:106157739 | NM_001127208.3 | c.2640del | p.His880Glnfs*41 | 16% | Likely Pathogenic | Tier 1A |
| TET2 | chr4:106155694 | NM_001127208.3 | c.598_601del | p.Leu200Lysfs*6 | 3% | Likely Pathogenic | Tier 3 |
| SOCS1 | chr16:11348883 | NM_003745.2 | c.446_453de | p.Glu149Glyfs*? | 13% | Likely Pathogenic | Tier 3 |
| **Pt ID: 79 year old male with ALK negative ALCL** | | | | | | | |
| PIK3R1 | chr5:67589620 | NM_181523.3 | c.1390_1410del  GATAGATTATA  TGAAGAATAT | p.Asp464_Tyr470del | 36% | Likely Pathogenic | Tier 2C |
| ANKRD11 | chr16:89346679 | NM_013275.6 | c.6271C>T | p.Gln2091* | 62% | Likely Pathogenic | Tier 3 |
| MSC | chr8:72756068 | NM_005098.4 | c.346G>A | p.Glu116Lys | 43% | Likely Pathogenic | Tier 3 |
| ***Pt ID: 39 year old male with PTCL, NOS *(Structural Variant: Fusion)** | | | | | | | |
| FYN::TRAF3IP2  e12::e2 | chr6:112015578 -  chr6:1119132971 | NM_002037.5 -  NM_147686.4 | - | - | 42 reads | Likely Pathogenic | Tier 3 |
| **Pt ID: 77 year old female with AITL** | | | | | | | |
| RHOA | chr3:49412973 | NM_001664.4 | c.50G>T | p.Gly17Val | 7% | Pathogenic | Tier 1A |
| CTNNB1 | chr3:41266124 | NM_001904.4 | c.121A>G | p.Thr41Ala | 6% | Pathogenic | Tier 2C |
| DNMT3A | chr2:25457242 | NM_022552.5 | c.2645G>A | p.Arg882His | 3% | Pathogenic | Tier 2D |
| DNMT3A | chr2:25463191 | NM_022552.5 | c.2302G>C | p.Asp768His | 27% | Likely Pathogenic | Tier 3 |
| TET2 | chr4:106164068 | NM_001127208.3 | c.3578G>A | p.Cys1193Tyr | 19% | Likely Pathogenic | Tier 3 |
| **Pt ID: 48 year old male with High grade B-cell lymphoma, double hit** | | | | | | | |
| CREBBP | chr16:3781324 | NM_004380.3 | c.5039_5041delC  CT | p.Ser1680del | 96% | Pathogenic | Tier 2C |
| TP53 | chr17:7578406 | NM_000546.6 | c.524G>A | p.Arg175His | 98% | Pathogenic | Tier 2C |
| SRSF2 | chr17:74732959 | NM_001195427.2 | c.284C>T | p.Pro95Leu | 43% | Pathogenic | Tier 2D |
| ARID1A | chr1:27057820 | NM_006015.6 | c.1528C>T | p.Gln510* | 38% | Likely Pathogenic | Tier 2C |
| CCND3 | chr6:41903710 | NM_001760.5 | c.847A>G | p.Thr283Ala | 42% | Likely Pathogenic | Tier 3 |
| KMT2D | chr12:49446989 | NM_003482.4 | c.954+1G>T | - | 60% | Likely Pathogenic | Tier 3 |
| STAT6 | chr12:57493830 | NM_003153.5 | c.1556A>G | p.Asp519Gly | 45% | Likely Pathogenic | Tier 3 |
| TBL1XR1 | chr3:176765130 | NM_024665.7 | c.822G>A | p.Trp274* | 48% | Likely Pathogenic | Tier 3 |
| **Pt ID: 55 year old male with High grade B-cell lymphoma, double hit** | | | | | | | |
| PHF6 | chrX:133551267 | NM_001015877.2 | c.903C>A | p.Tyr301* | 90% | Pathogenic | Tier 3 |

**Figure S3:** **Adverse Events (AEs).**

A). Adverse Events per Patient (N=8):

B). Adverse Events per Cycle (N=30):


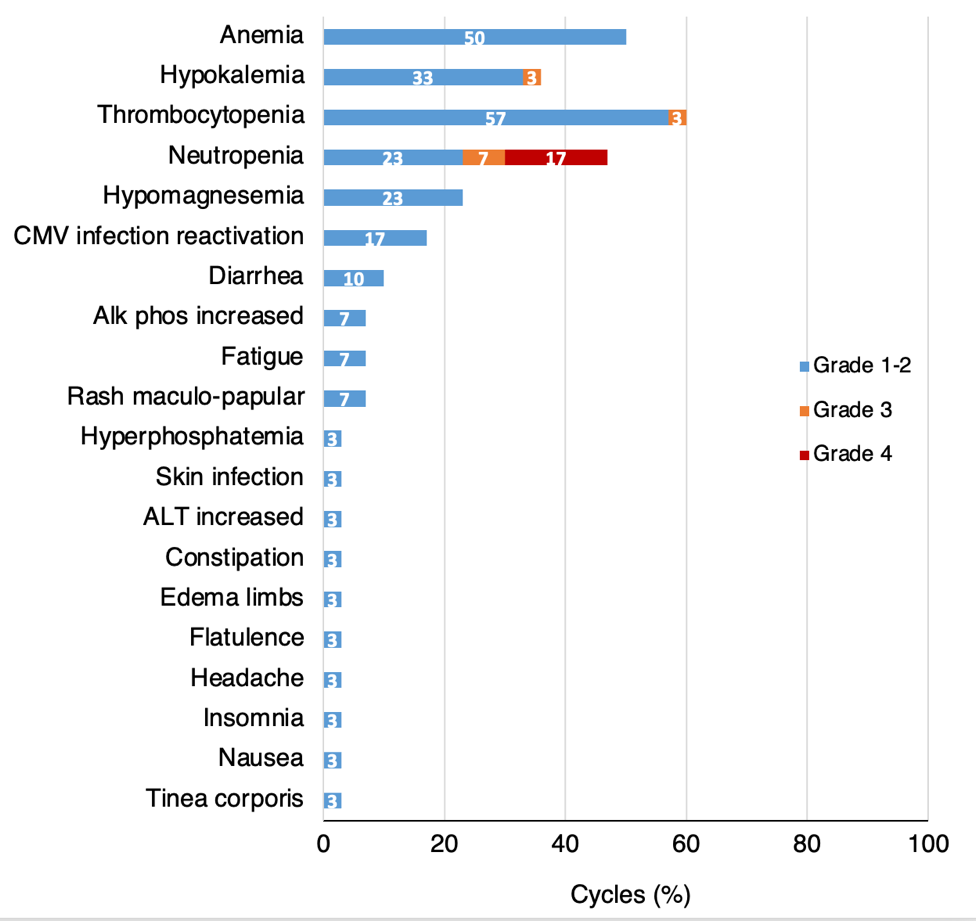


**Figure S4: Progression-free Survival (PFS) and Overall Survival (OS) in All Patients.**

**Supplemental references**

16. Melani C, Lakhotia R, Pittaluga S, Phelan JD, Huang DW, Wright G, et al. Combination Targeted Therapy in Relapsed Diffuse Large B-Cell Lymphoma. New England Journal of Medicine. 2024;390(23):2143-55.

17. Cheson BD, Fisher RI, Barrington SF, Cavalli F, Schwartz LH, Zucca E, et al. Recommendations for initial evaluation, staging, and response assessment of Hodgkin and non-Hodgkin lymphoma: the Lugano classification. Journal of clinical oncology : official journal of the American Society of Clinical Oncology. 2014;32(27):3059-68.

18. Xie S, Wei F, Sun YM, Gao YL, Pan LL, Tan MJ, et al. EZH2 inhibitors abrogate upregulation of trimethylation of H3K27 by CDK9 inhibitors and potentiate its activity against diffuse large B-cell lymphoma. Haematologica. 2020;105(4):1021-31.
